# Eco-Friendly Surfactants Based on Fatty Acids and Monoethanolamine for Efficient Oil Spill Remediation

**DOI:** 10.1021/acsomega.5c06734

**Published:** 2025-09-26

**Authors:** Sádwa F. Ribeiro, R. S. Santiago-Aguiar

**Affiliations:** Chemical Engineering Department, 28121 Federal University of Ceara, Pici Campus, Bl. 709,Fortaleza, Ceara 60440-554, Brazil

## Abstract

Environmental concerns surrounding the use of toxic chemical dispersants in oil spill remediation have prompted the development of safer, more sustainable alternatives. Surfactants derived from fatty acids offer advantages such as low toxicity, biocompatibility, and cost-effectiveness. In this study, two novel surfactants based on monoethanolamine/lauric acid (MEA-LA) and monoethanolamine/oleic acid (MEA-OA) were evaluated for their potential in oil spill cleanup applications. To this end, measurements of surface tension, emulsification index, crude oil dispersion, and motor oil removal from sand were conducted. MEA-LA and MEA-OA reduced surface tension to 21.7 ± 0.03 mN/m and 25.2 ± 0.00 mN/m, with CMC values of 8.5 and 1.2 mmol/L, respectively. Emulsification assays revealed stable emulsions after 24 h, with indices of 52.6% ± 1.0 (motor oil) and 74.4% ± 2.0 (kerosene) for MEA-LA and 50% ± 2.0 (motor oil) and 67.1% ± 3.0 (kerosene) for MEA-OA, consistently outperforming sodium dodecyl sulfate (SDS). Both surfactants demonstrated hydrocarbon removal from sand, achieving efficiencies of 57.03% ± 0.03 (MEA-LA) and 61.67% ± 0.05 (MEA-OA) at 3 × CMC. In addition, low-energy dispersion confirmed their ability to produce fine, stable oil droplets, favoring biodegradation. Acute toxicity assays with *Artemia salina* showed lethal concentration values (LC_5__0_) of 2057 μg/mL (MEA-LA) and 1956 μg/mL (MEA-OA), classifying both as nontoxic. These results suggest that fatty acid-based surfactants are promising candidates for replacing conventional dispersants in oil spill response, contributing to the reduction of environmental and socioeconomic impacts.

## Graphical abstract

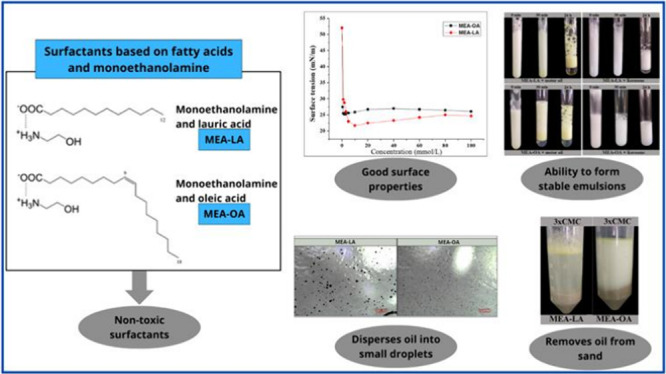

## 1 Introduction

Various activities in oil exploration, production, and transportation to meet global energy demands have increased the incidence of marine spills. These incidents, often occurring during maritime transport, whether accidental or intentional, release toxic petroleum hydrocarbons that not only devastate marine ecosystems but also harm fisheries, tourism, and human health, since the compounds present in oil have high toxicity.^1,2^

To mitigate these impacts, a range of spill-response techniques has been developed, including mechanical recovery (booms and skimmers), in situ burning, absorbents, and chemical surfactants.^3^ The choice of method depends on factors such as oil properties, spill volume, and environmental conditions.^4^ Among these, chemical surfactants reduce oil–water interfacial tension, breaking slicks into fine droplets that enhance microbial degradation.^5,6^ However, many conventional formulations exhibit high toxicity at low concentrations and persist in the environment.^7,8^ For instance, during the 2010 Deepwater Horizon disaster, approximately 7000 m^3^ of Corexit 9500A were applied, leading to long-term contamination of deep-water habitats and documented toxicity to coral endosymbionts, crustaceans, seabirds, and marine mammals.^8–11^

Growing environmental regulations and public demand for sustainable, less-impactful remediation strategies have driven researchers and industry to explore biodegradable surfactants with low ecotoxicity.^12,13^ Among these, fatty-acid–based surfactants have garnered significant interest due to their renewable feedstocks, affordability (being readily sourced from plant and animal lipids), intrinsic biodegradability, and favorable biocompatibility profiles.^14,15^ Additionally, recent studies have demonstrated that surfactants produced from fatty acids can exhibit superior characteristics compared to conventional petroleum-derived surfactants, such as improved surface and interfacial properties.^16–18^ For example, Ali et al.,^19^ synthesized surfactants from oleic, linoleic, and erucic acids, achieving critical micelle concentrations (CMCs) of 1.7 mM, 2.0 mM, and 0.8 mM, respectively, substantially below values for conventional benchmarks such as sodium dodecyl sulfate (SDS) (7.8 mM) and sodium dodecylbenzenesulfonate (SDBS) (2.9 mM). Similarly, Saxena et al.^20^ reported a palm-oil–derived surfactant that reduced surface tension from 72 to 32.5 mN/m and decreased carbonate–oil wettability from 82.5° to 20.7° within 1000 s, facilitating oil removal. Nazar et al.^21^ also reported excellent surface-active properties of an ionic liquid surfactant synthesized from choline chloride and myristic acid [Cho]­[Mys]. When combined with Span 80, it exhibited oil dispersion efficiency exceeding 80%.^22^

Recent research has shown that oleic- and linoleic-acid derivatives can achieve critical micelle concentrations (CMC) below 2 mM, outperforming benchmarks like SDS, and that ionic liquids also emerge as promising eco-friendly alternatives for oil remediation in marine waters.^23,18^ Despite these advances, the poor water solubility of long-chain fatty acids limits practical application. Incorporation of organic counterions such as monoethanolamine (MEA) prevents fatty-acid crystallization and enhances aqueous solubility and micelle formation.^17,24^ Yet, despite MEA’s low cost and good thermal stability, systematic evaluations of MEA-based surfactants under realistic oil-spill conditions remain scarce.

In this context, the main objectives of this study were: (i) to synthesize two novel MEA-derived surfactants and evaluate their potential for oil spill remediation; (ii) to determine their surface-active properties, including surface tension reduction and critical micelle concentration (CMC); (iii) to assess emulsion stability and crude oil dispersion under low-energy conditions; (iv) to evaluate hydrocarbon removal efficiency from sand; and (v) to determine acute toxicity through *Artemia salina* bioassays.

## 2 Materials Synthesis, and Characterization Methods

### 2.1 Materials

Lauric acid (≥98%) was purchased from Êxodo Científica Ltd. (Brazil); oleic acid (90%) and sodium dodecyl sulfate (SDS, ≥ 99%) from Sigma-Aldrich (United States); and monoethanolamine (≥99%) from Neon Comercial Reagentes Analíticos Ltd. (Brazil). Quartz sand (50–70 mesh) was obtained from Sigma-Aldrich (United States). Motor oil and *Artemia salina* cysts were sourced locally in Fortaleza, Ceara, Brazil. Medium crude oil (24 °API; properties in Table S1 – Supporting Information) was supplied by the Phase Equilibrium Laboratory, Chemical Engineering Department, Federal University of Ceara.

### 2.2 Surfactant Synthesis

Surfactant solutions (MEA-LA and MEA-OA) were prepared following Lu et al.^25^ by mixing equimolar amounts of monoethanolamine and the corresponding fatty acid (lauric acid C_1__2_H_2__4_O_2_ or oleic acid C_1__8_H_3__4_O_2_) in deionized water as solvent. The mixtures were sonicated at 318.15 K for 5 h in an Ultrasonic bath SSBu (SolidSteel, Brazil) and subsequently stirred at 300 rpm for 48 h at room temperature. Chemical structures and abbreviations are summarized in Table 1.

**Table 1:**
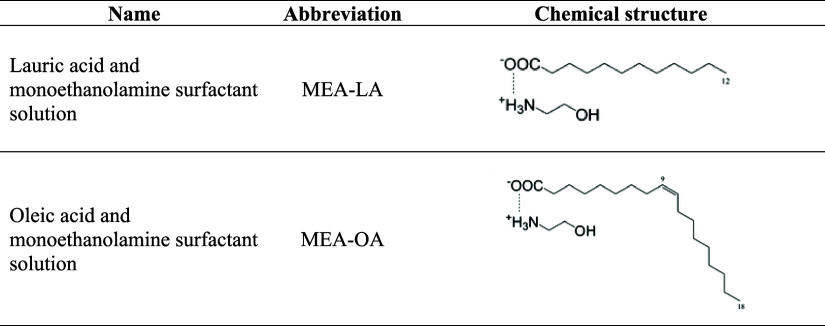
Prepared Surfactant Solutions

### 2.3 Surface Tension and Critical Micellar Concentration (CMC)

Surface tension was measured by the Du Nöuy ring method^26^ using a Krüss EasyDyne K-20 tensiometer (Krüss, Germany) with a Thermostatic bath F25-ED (Julabo, Germany) at 303.15 K. Surfactant concentrations ranged from 0.5 to 100 mmol L^–^^1^, with 15 replicates per measurement. Instrument uncertainties are ± 0.01 mN m^–^^1^ and ± 0.01 K. Calibration with toluene at 293.15 K yielded 30 mN m^–^^1^, consistent with literature values. CMC values were determined from the breakpoint in surface tension vs log-concentration plots (Figures S1–S2, Supporting Information).^27^

It is important to note that the surface tension measured for the deionized water control under our experimental conditions was 52.0 mN m^–^^1^, which is lower than the nominal value for pure water (∼72 mN m^–^^1^ at room temperature). This difference can be attributed to the sensitivity of surface tension to trace impurities, dissolved gases, and handling conditions. For this reason, all reductions reported in this work are expressed relative to the experimentally measured control baseline rather than the tabulated value for pure water.

### 2.4 Emulsification Index

Emulsification capacity was assessed by mixing 2 mL of surfactant solution (100 mmol L^–^^1^) with 2 mL of motor oil or kerosene in a Vortex mixer Q220 (Quimis, Brazil) (2 min at maximum speed). Samples rested at 298.15 K and emulsified layer heights were recorded after 30 min and 24 h to check stability. The emulsification index (E_24_) was calculated in triplicate after 24 h by the ratio between the height of the emulsified layer and the total height of the mixture^28^ as eq 1:

$$E_{24}=\frac{h_{emulsion}}{h_{total}}x100\%$$ 1

### 2.5 Motor Oil Removal from Sand

As described by Chaprão et al.^29^ with modifications, the procedure for removing motor oil from sand was carried out by impregnating the sand with motor oil in a proportion of 1:10 (m/m) in 50 mL Falcon tubes. To wash the sand, 20 mL of the surfactant solutions MEA-LA and MEA-OA were added at 1/2 the CMC, at the CMC, and at three times the CMC. Sodium dodecyl sulfate (SDS) and water were used as experimental controls, and the analysis was performed in duplicate. Tubes were shaken horizontally in a Dubnoff bath Q226 M (Quimis, Brazil) at 303.15 K (150 cycles/min) for 24 h. After this period, the washing surfactant solution containing the extracted oil was removed and the sand containing the residual oil was dried in an oven at 333.15 K for 48 h. The quantification of the oil removed from the sand was carried out gravimetrically by comparing it to the mass of the sand before impregnation with oil.

### 2.6 Oil Displacement Area (ODA)

To determine the oil displacement area, the method described by Pele et al.^30^ was adapted. In this sense, 1 mL of medium crude oil (24 °API) was spread on 40 mL of deionized water in a 63.3 cm^2^ Petri dish at 343.15 K. After uniform spreading, 0.5 mL of surfactant (100 mmol L^–^^1^), SDS (100 mmol L^–^^1^), or water (control) was added at the center. Experiments were performed in triplicate, and the diameters of the clear zones were measured with a ruler. Statistical analysis of the data was performed using Microsoft Excel. The mean values were used to calculate the oil displacement area (ODA) according to eq 2, given by Morikawa et al.:^31^

$$\mathrm{ODA}=\pi \times (radius{)}^{2}$$ 2

### 2.7 Low Energy Dispersion Test

Based on Fernandes et al.,^32^ a low-energy dispersion test was performed by adding 100 μL of crude oil (24 °API) to the surface of 100 mL of MEA surfactant solutions (100 mmol/L) in Erlenmeyer flasks. Samples were shaken at 250 rpm and 303.15 K for 24 h in an Orbital shaker TE-424 (Tecnal, Brazil) incubator. After 10 min of settling, an aliquot of the solution was examined using the Digital Microscope (HAIZ, China) (50x-1600x) with HiView software v1.4 to visualize the dispersed oil droplets.

### 2.8 Acute Toxicity against the Microcrustacean *Artemia salina*

Toxicity assays were performed following Miguel et al.^33^ and Meyer et al.^34^ *Artemia Salina* cysts (0.2 g) were hatched in 200 mL of 3.5% NaCl (pH 8) under light and aeration at 297.15 K for 48 h and subsequently separated by positive phototaxis. Ten nauplii were exposed to five concentrations of the surfactants MEA-LA and MEA-OA (10, 100, 250, 500, and 1000 μg/mL) in 24-well plates, to the positive control (aqueous NaCl solution at 3.5% m/v and pH 8), and the negative control, which consisted of a 0.5 mol/L potassium dichromate solution. Mortality was recorded at 24 and 48 h of incubation under constant lighting. The analysis was performed in triplicate and was only validated when the survival rate of the positive control group was ≥ 90%.^35^ The percentage of deaths was determined and converted into probit values using the Microsoft Excel program to obtain the concentration that killed 50% of the individuals after 48 h (LC_50_).

## 3 Results and Discussion

This section presents the physicochemical characterization of MEA-LA and MEA-OA surfactants and evaluates their performance in oil-spill remediation tests. We first analyze surface tension and determine the critical micellar concentration (CMC), then assess emulsification, followed by kinetic removal of motor oil from sand, oil displacement area (ODA), low-energy dispersion, and acute toxicity against *Artemia salina.*

### 3.1 Surface Tension and Critical Micellar Concentration

The interfacial activity of a surfactant is gauged by its ability to adsorb at the oil–water interface and lower surface tension.^36^ Figure 1 illustrates the surface tension of MEA-LA and MEA-OA as a function of concentration at 303.15 K. Both surfactants substantially reduced the surface tension of the water control (52.0 mN/m under our experimental conditions) to minimum values of 21.7 ± 0.03 mN/m (MEA-LA) and 25.2 ± 0.00 mN/m (MEA-OA), corresponding to reductions of approximately **58%** and **52%**, respectively. The ability to reduce surface tension is important in oil spill remediation, as it facilitates the dispersion and stabilization of small droplets, aiding the remediation process.^37,38^ These minima are superior to those of common dispersants and surfactants reported in the literature: Corexit 9500A (39.96 mN/m at 293.15 K),^39^ sodium dodecyl sulfate (SDS) (32.43 mN/m at 308.15 K),^40^ and sodium dodecylbenzenesulfate (SDBS) (32.6 mN/m at 298.15 K).^41^

**Figure 1:**
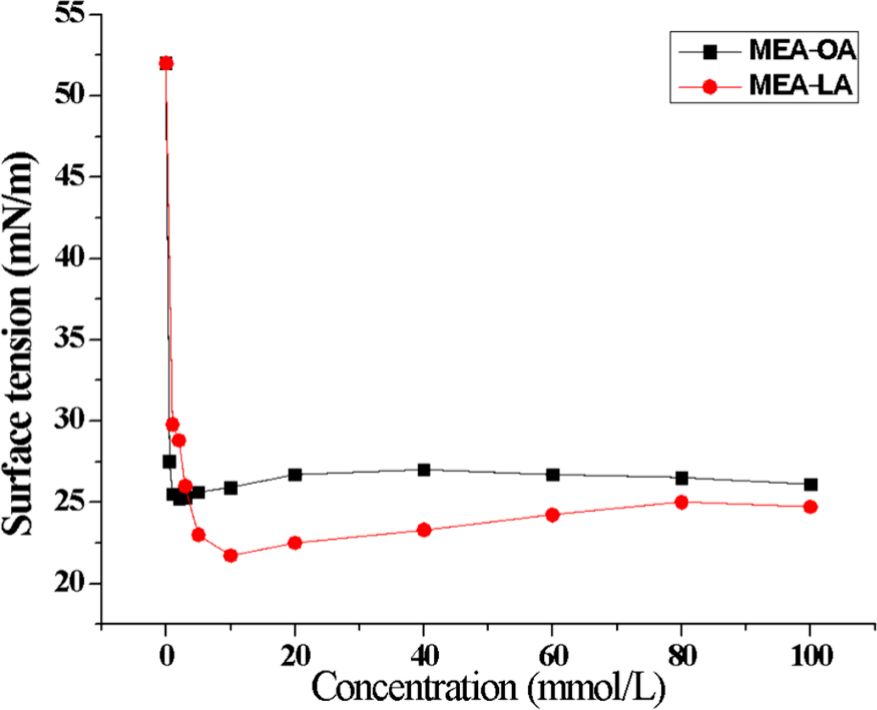
Surface tension of MEA-OA and MEA-LA in deionized water at 303.15 K at different concentrations

Surface tension data also reveal the onset of micelle formation, that is, the concentration at which surfactant solutions begin to form micelles in large quantities and the tension is no longer significantly reduced as the surfactant concentration increases.^42^ The CMC of MEA-LA and MEA-OA was determined by the intersection of linear fits to the pre- and postmicellar regions (Figures S1–S2). As summarized in Table 2, MEA-OA exhibits a lower CMC (1.2 mmol/L) than MEA-LA (8.5 mmol/L), indicating higher efficiency (lower concentration required), whereas MEA-LA is more effective in minimizing surface tension. According to Vaz *et a*l.,^43^ there is a distinction between an effective and an efficient surfactant. Efficacy refers to the minimum surface tension that can be achieved, while efficiency is evaluated by the surfactant concentration required to significantly reduce the surface tension of water, that is, the CMC. Because of this, among the surfactants studied, MEA-OA is the most efficient, as it has the lowest CMC, while MEA-LA is the most effective since it reached a lower surface tension value.

**Table 2:** CMC and Minimum Surface Tension of Surfactants MEA-LA and MEA-OA at 303.15 K^a^

Surfactant	CMC (mmol/L)	Minimum Surface Tension (mN/m)
MEA-LA	8.5	21.7 ± 0.03
MEA-OA	1.2	25.2 ± 0.00

a Instrument uncertainties: ± 0.01 mN m^–^^1^ (surface tension) and ± 0.01 K (temperature).

The lower CMC of MEA-OA compared to conventional surfactants (e.g., Tween 20:2.4 mmol/L; Tween 80:2.5 mmol/L; SDS: 8.1 mmol/L) underscores its potential to reduce the required dispersant volume and associated environmental load.^44,45^ This is an important parameter when considering applications in oil spill remediation, as one major concern is the volume of dispersant and solvent introduced into the marine ecosystem, which carries economic and environmental consequences.^46^ Therefore, surfactants capable of significantly reducing surface tension at lower concentrations are more advantageous.

### 3.2 Emulsification Index

The emulsification index is a key property for surfactants in oil-spill remediation, as stable emulsions enhance oil dispersion by increasing interfacial area.^47^ Emulsions of MEA-LA and MEA-OA (100 mmol/L) with kerosene or motor oil were prepared by vortexing (2 min) and observed at 0, 30 min, and 24 h (Figure 2). Immediately after vortexing, both hydrocarbons formed milky emulsions. After 30 min, MEA-LA emulsions remained stable for both kerosene and motor oil, while MEA-OA/motor oil emulsions showed phase separation, forming an upper oil-rich layer and a lower aqueous layer. This behavior indicates that emulsion stability depends on the hydrocarbon type and surfactant structure.

**Figure 2:**
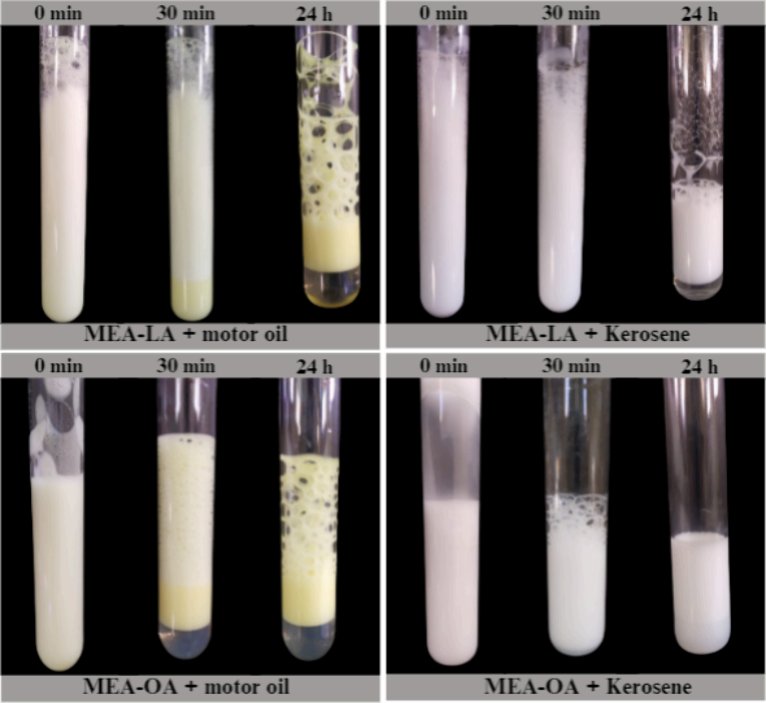
Emulsions formed by MEA-LA and MEA-OA with motor oil and kerosene at 0 min, 30 min, and 24 h

According to Sousa et al.,^48^ an emulsion is considered stable if it retains 50% or more of its original volume after 24 h. Emulsification index (E_24_) after 24 h (Figure 3) demonstrates that both MEA-LA and MEA-OA produce stable emulsions (E_24_ ≥ 50%) with kerosene and motor oil, outperforming SDS under identical conditions. Furthermore, the values obtained in this study were significantly higher than those reported by Nwaguma et al.,^49^ who observed an emulsification index (E_24_) of approximately 20% for kerosene using a biosurfactant produced by the IVN51 strain of *Klebsiella pneumoniae*. The higher E_24_ values for kerosene versus motor oil reflect greater affinity for lower-molecular-weight hydrocarbons. Stable emulsions are advantageous for spill remediation, as they enhance microbial biodegradation by expanding the oil–water interfacial area.^5^

**Figure 3:**
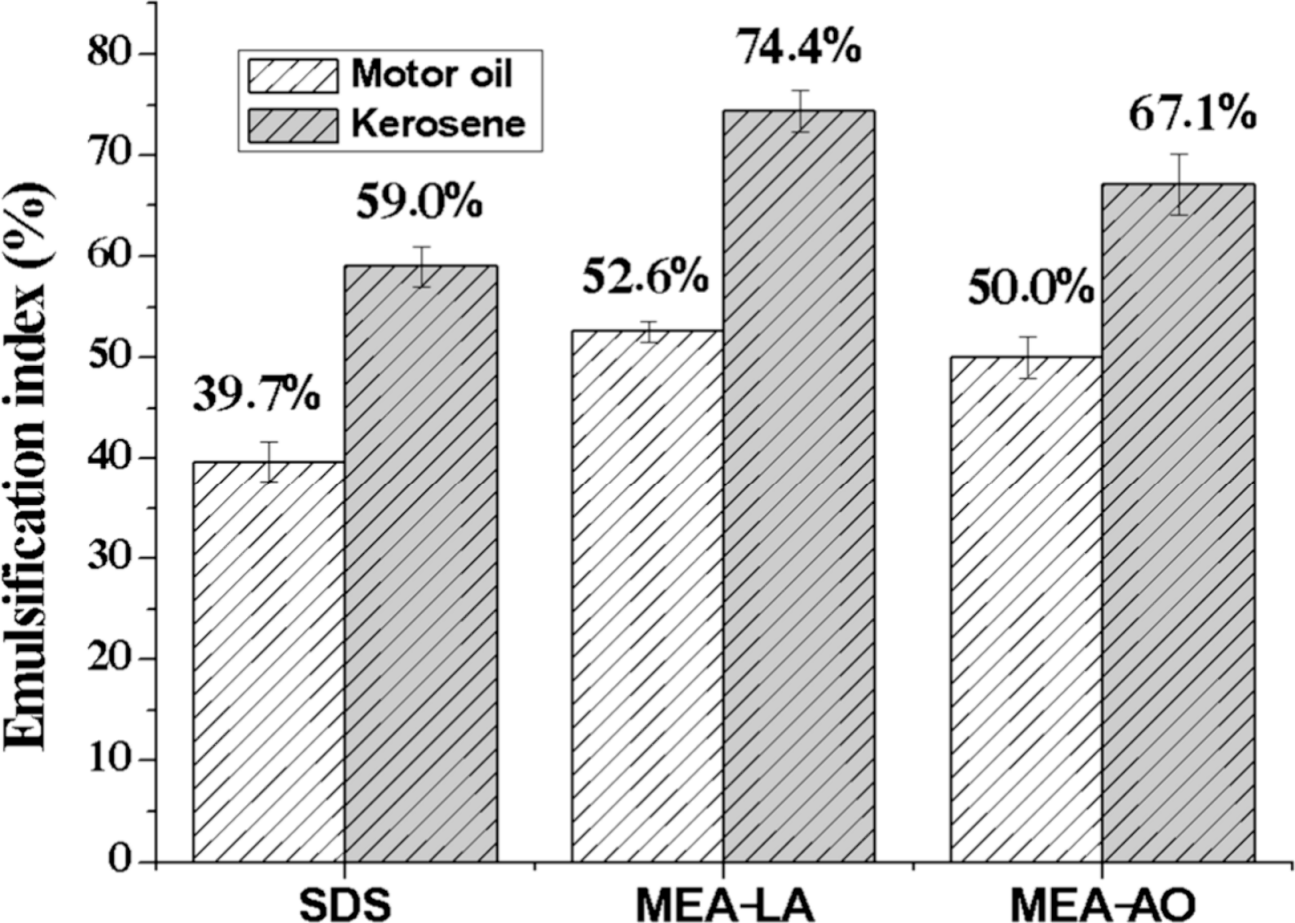
Emulsification index (E_24_) of MEA-LA, MEA-OA, and SDS with kerosene and motor oil at 100 mmol L^–^^1^.

### 3.3 Removal of Motor Oil from Sand (Kinetic Testing)

Coastal contamination from oil settling into sediments poses remediation challenges due to tidal and wave action.^50^ Surfactant washing can mobilize and solubilize hydrophobic contaminants in sand.^51^ We assessed motor oil removal using MEA-LA and MEA-OA at 0.5× CMC, 1× CMC, and 3× CMC over 24 h at 303.15 K. SDS and water served as controls.

The results presented in Figure 4 demonstrate that the surfactants MEA-LA and MEA-OA are useful for remediating soil contaminated with hydrophobic compounds. When used with triple the critical micellar concentration, they achieved removal percentages of 57.03% ± 0.03 and 61.67% ± 0.05, respectively, within 24 h. The higher oil removal rates at the highest concentration can be explained by the solubilization mechanism. Specifically, the solubility of the oil increases drastically due to the aggregation of the surfactant micelles, characterized by the hydrophilic external part and the hydrophobic internal part.^52^ Fatty acid–based surfactants recovered about 20 times more oil from the sand than the control (2.64% ± 0.01). Furthermore, the performance (3× CMC) exceeded that of the bioemulsifier produced by A. *venetianus* AMO1502, evaluated by D’Almeida,^53^ which removed only approximately 25% of motor oil from sand after 7 days of agitation.

**Figure 4:**
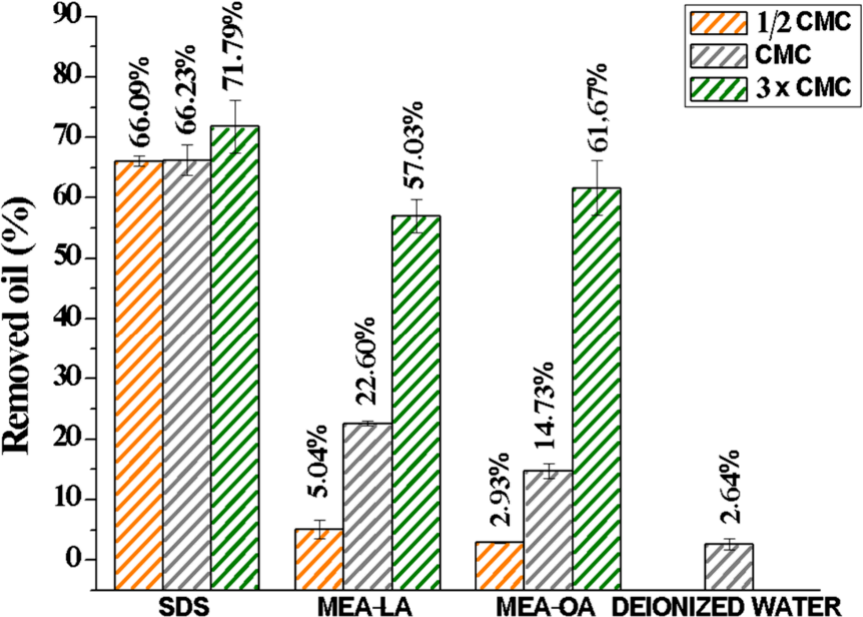
Percentage of motor oil removed from sand after 24 h washing with MEA-LA, MEA-OA, and SDS at various multiples of their CMC.

SDS exhibited high removal even at 0.5× and 1× CMC, indicating a predominant mobilization mechanism via reduced capillary forces and altered sand wettability, facilitating oil droplet release.^54^

Considering that both lauric and oleic acids are widely available from plant-based industrial sources (e.g., coconut oil, palm oil, soybean oil), and that MEA is commercially abundant, these surfactants are amenable to large-scale synthesis at low cost using existing infrastructure. Compared to SDS or Corexit-type formulations, which often require cosolvents or dispersing agents and exhibit significant ecotoxicity, the MEA-based surfactants presented here achieve similar or superior performance with a markedly reduced environmental and economic burden.

### 3.4 Oil Displacement Area

The oil displacement area (ODA) test evaluates a surfactant’s ability to spread and thin an oil slick by forming a clear zone on the oil-covered water surface.^55,56^ Using a Petri dish of total area 63.6 cm^2^, 0.5 mL of MEA-LA or MEA-OA (100 mmol L^–^^1^) was added to the center of a 1 mL oil film (crude oil, 24 °API, or motor oil) on water at 343.15 K. The percentage clear-zone area was calculated from measured diameters (Figure 5).

**Figure 5:**
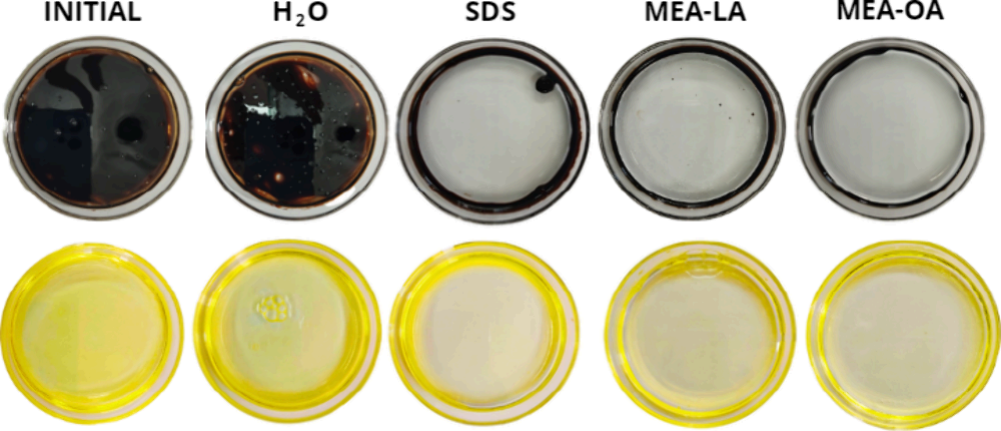
Oil displacement area (%) of crude oil (24 °API) and motor oil after addition of MEA-LA, MEA-OA, and SDS at 100 mmol L^–^^1^.

For crude oil, MEA-LA and MEA-OA achieved clear-zone percentages of 87.2% ± 1.0 and 95.7% ± 1.0, respectively, demonstrating excellent dispersion capacity. For motor oil, clear-zone percentages were 89.3% ± 1.2 (MEA-LA) and 95.7% ± 1.0 (MEA-OA). Both fatty-acid surfactants outperformed SDS, which displaced 73.2% ± 1.1 of motor oil and 75.2% ± 1.0 of crude oil under identical conditions. Similarly, Silva et al.^57^ evaluated the commercial surfactant Tween 80 and a biosurfactant produced by *Starmerella bombicola*, achieving oil displacement percentages of 74.7% and 73.3%, respectively, for crude oil.

### 3.5 Low-Energy Dispersion Test

Low-energy dispersion tests evaluate a surfactant’s ability to break an oil slick into fine droplets under gentle agitation.^32^ Figure 6 presents microscopic images of crude oil (24 °API) dispersions in aqueous MEA-LA and MEA-OA solutions (100 mmol L^–^^1^) after shaking for 24 h at 250 rpm and 303.15 K.

**Figure 6:**
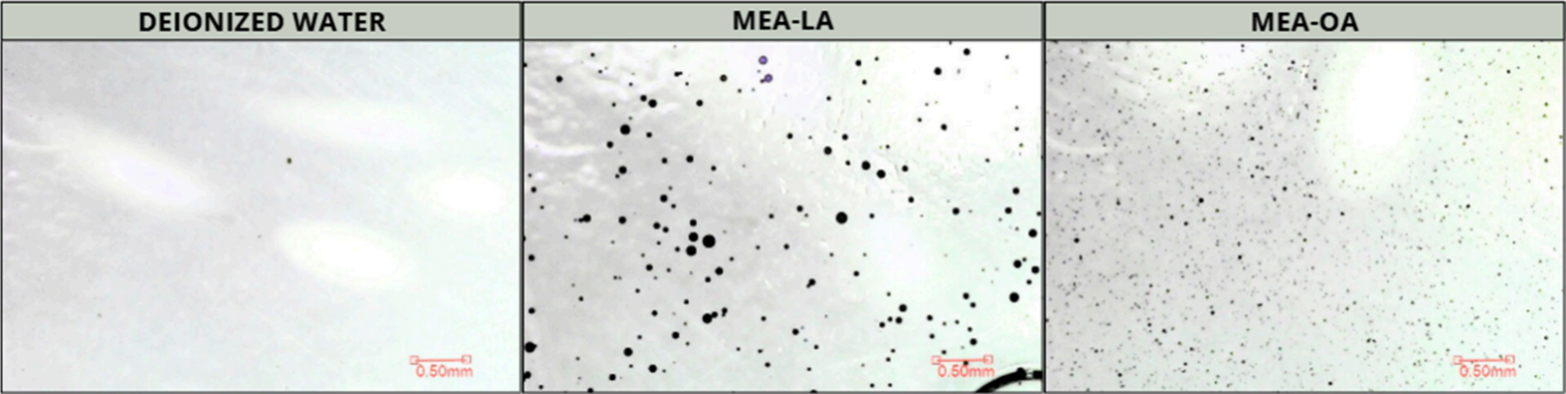
Microscopic images of crude oil droplets dispersed by MEA-LA and MEA-OA (100 mmol L^–^^1^) in the low-energy dispersion test.

Both fatty-acid surfactants effectively dispersed the oil into small droplets, enhancing the surface area available for microbial degradation.^58,59^ Similar behavior was reported in a previous study that evaluated the dispersion capacity of a fatty acid–based ionic liquid surfactant, choline laurate.^18^

MEA-OA produced notably smaller and more numerous droplets than MEA-LA, indicating reduced coalescence and higher dispersion stability. This behavior may result from MEA-OA’s faster adsorption at the oil–water interface and stronger hydrophobic interactions due to its unsaturated C18 chain, leading to lower interfacial tension and more robust droplet stabilization.^60^

### 3.6 Acute Toxicity to *Artemia salina*

Large-scale spill remediation often requires substantial dispersant volumes, raising concerns due to the high toxicity of conventional formulations.^61^ The toxicity of MEA-LA and MEA-OA against *Artemia salina* nauplii was evaluated over 48 h. Nauplii were exposed to surfactant concentrations of 10, 100, 250, 500, and 1000 μg/mL, with mortality recorded at 24 and 48 h (Figure 7).

**Figure 7:**
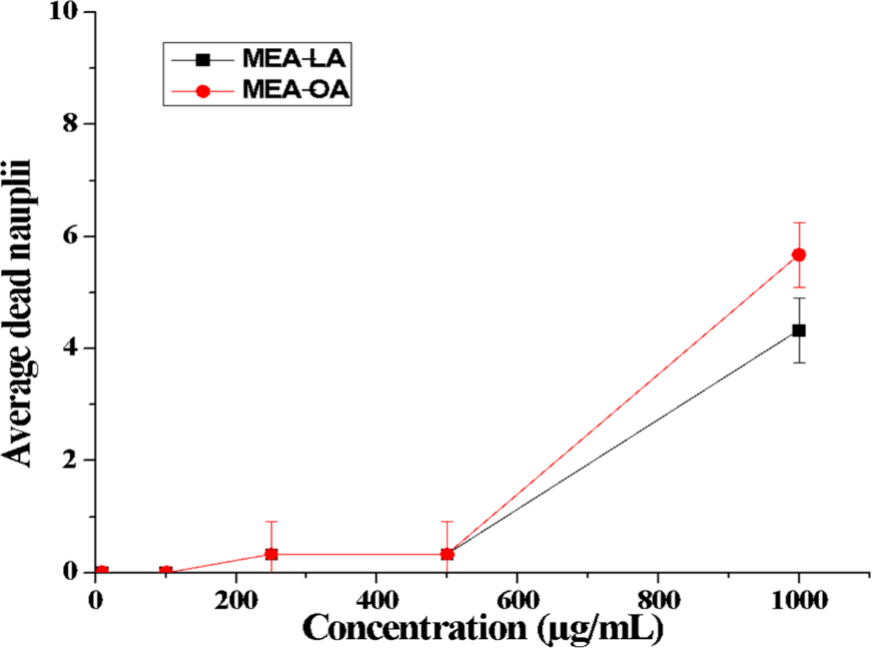
Percentage mortality of *Artemia salina* nauplii after 48 h exposure to MEA-LA and MEA-OA.

At 48 h, no mortality occurred at 10 or 100 μg/mL for either surfactant. Mortality at 250 and 500 μg/mL was only 3.3% for both MEA-LA and MEA-OA. At 1000 μg/mL, mortality increased to 43.3% (MEA-LA) and 56.7% (MEA-OA).

LC_5__0_ values calculated via probit analysis are presented in Table 3. Both surfactants exceed the EU Directive 93/67/EEC threshold for nontoxic substances (LC_5__0_ > 100 μg/mL),^62^ indicating low acute toxicity. In comparison, Corexit 9527 (LC_5__0_: 53–84 μg/mL) and Corexit 9500 (LC_5__0_: 21 μg/mL) are classified as highly toxic.^63,64^ The slightly higher LC_5__0_ for MEA-LA (2057 μg/mL) versus MEA-OA (1956 μg/mL) may be related to differences in molecular weight and hydrophobic chain saturation.^65^

**Table 3:** LC_5__0_ Values for MEA-LA and MEA-OA against *Artemia salina* after 48 h

Surfactant	LC_50_ (μg/mL)^a^
MEA-LA	2057
MEA-OA	1956

a Uncertainty of LC_5__0_ determinations: 95% confidence interval (±10–20%).

The surfactants developed in this study align with multiple Principles of Green Chemistry, particularly the use of renewable feedstocks (fatty acids from plant oils; Principle 7), the design of safer chemicals with low acute toxicity (Principle 4), and the use of mild energy conditions (Principle 6), as the synthesis was conducted under moderate temperature and aqueous conditions. Moreover, the method avoids the use of hazardous organic solvents and minimizes synthetic complexity (Principle 1). Given that lauric and oleic acids are abundant, low-cost, and industrially available, and that monoethanolamine is widely used in pharmaceutical and surfactant production, the scalability and economic feasibility of these formulations are highly promising. Although direct biodegradation testing was beyond the scope of this work, the chemical structures of MEA-LA and MEA-OA, composed of a linear fatty acid chain and a primary alkanolamine, are similar to known biodegradable surfactants, and are expected to undergo microbial degradation via β-oxidation and deamination pathways. These attributes reinforce the potential of MEA-based surfactants as environmentally benign and industrially scalable alternatives for oil spill response.

## 4 Conclusions

In this study, two novel surfactants based on fatty acids and monoethanolamine, MEA-LA and MEA-OA, were successfully synthesized and demonstrated strong surface activity (surface tension reduction to 21.7 ± 0.03 and 25.2 ± 0.00 mN/m; CMC of 8.5 and 1.2 mmol/L, respectively). Emulsification assays revealed stable emulsions after 24 h, with indices of 52.6% ± 1.0 (motor oil) and 74.4% ± 2.0 (kerosene) for MEA-LA, and 50% ± 2.0 (motor oil) and 67.1% ± 3.0 (kerosene) for MEA-OA, consistently outperforming sodium dodecyl sulfate (SDS). Furthermore, the surfactants were able to remove motor oil from sand, achieving removal percentages of 57.03% ± 0.03 (MEA-LA) and 61.67% ± 0.05 (MEA-OA) when applied at three times their CMC. High dispersion efficiency was also observed for crude oil, with clear-zone percentages of 87.2% ± 1.0 for MEA-LA and 95.7% ± 1.0 for MEA-OA, as well as for motor oil, with values of 89.3% ± 1.2 for MEA-LA and 95.7% ± 1.0 for MEA-OA. In addition, both surfactants promoted fine-droplet dispersion under low-energy conditions. Acute toxicity assays using *Artemia salina* indicated LC_5__0_ values of 2057 μg/mL (MEA-LA) and 1956 μg/mL (MEA-OA), classifying both surfactants as nontoxic under EU Directive 93/67/EEC. These findings underscore the potential of MEA-LA and MEA-OA as eco-friendly, cost-effective alternatives for oil-spill remediation that combine high efficacy at low concentrations with minimal environmental risk. Further work should focus on performance in saline and dynamic marine conditions, formulation optimization with cosurfactants, and pilot-scale field trials to validate real-world applicability.

## Supplementary Material



## Notes

Additional details for this study, including the determination of the critical micelle concentration (CMC) of the surfactant based on monoethanolamine and lauric acid (MEA–LA, Figure S1), the determination of the CMC of the surfactant based on monoethanolamine and oleic acid (MEA–OA, Figure S2), and the properties of the crude oil used in this study (Table S1) (PDF)
