# Supplementary material for: Eco-Friendly Surfactants Based on Fatty Acids and Monoethanolamine for Efficient Oil Spill Remediation
Source: ACS Omega. 2025 Sep 26;10(39):45957–64. doi: 10.1021/acsomega.5c06734 (PMC12509018; doi:10.1021/acsomega.5c06734)
Supplement: Supplementary file 1 [file ao5c06734_si_001.pdf]

Supporting Information

Eco-Friendly Surfactants Based on Fatty Acids and Monoethanolamine for Efficient Oil Spill Remediation

Sádwa F. Ribeiro[†], Rilvia S.Santiago-Aguiar[*†]

[†]Chemical Engineering Department, Federal University of Ceara, Fortaleza, Ceara, 60440-900, Brazil

[*]Corresponding Author:

Rílvia S. de Santiago-Aguiar, Chemical Engineering Department, Federal University of Ceará, Pici Campus, Bl. 709, 60440-900, Fortaleza - CE, Brazil; [*]email: rilvia@ufc.br; Tel: +55-85-33669611

List of supplementary materials:

- **Figure S1.** Determination of the critical micelle concentration (CMC) of the surfactant based on monoethanolamine and lauric acid (MEA-LA).

- **Figure S2.** Determination of the critical micelle (CMC) concentration of the surfactant based on monoethanolamine and oleic acid (MEA-OA)

- **Table S1.** Properties of crude oil used in this study.

Figure S1. Determination of the critical micelle concentration (CMC) of the surfactant based on monoethanolamine and lauric acid (MEA-LA).

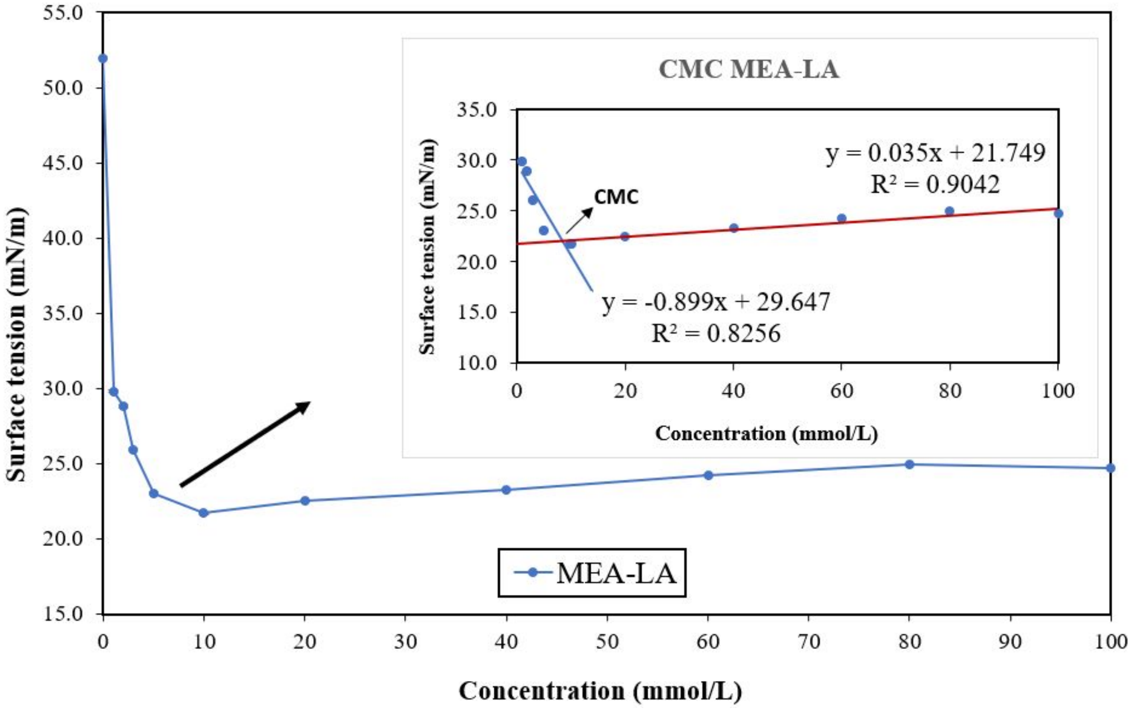

Figure S2. Determination of the critical micelle (CMC) concentration of the surfactant based on monoethanolamine and oleic acid (MEA-OA)

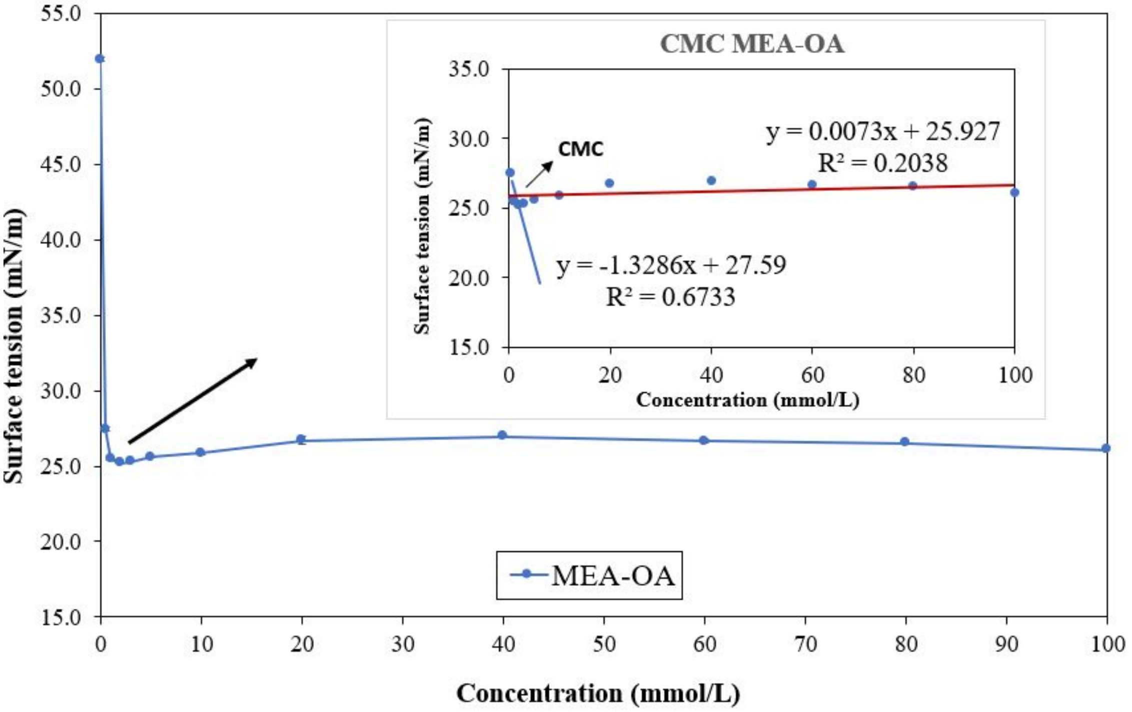

Table S1. Properties of crude oil used in this study.

Temperature (K)	Density ($g.cm^{-3}$)	Dynamic viscosity (m.Pa.s)	Kinematic viscosity ($mm^2.s^{-1}$)
293.15	0.9019	1095.9	1215.0
313.15	0.8865	57.064	64.373
333.15	0.8730	24.310	27.847
353.15	0.8597	12.782	14.868

Measurement uncertainties: 0.35% for viscosity and 0.0005 g/cm^3 for density.
